# SPAG5 upregulation predicts poor prognosis in cervical cancer patients and alters sensitivity to taxol treatment via the mTOR signaling pathway

**DOI:** 10.1038/cddis.2014.222

**Published:** 2014-05-22

**Authors:** L-J Yuan, J-D Li, L Zhang, J-H Wang, T Wan, Y Zhou, H Tu, J-P Yun, R-Z Luo, W-H Jia, M Zheng

**Affiliations:** 1 State Key Laboratory of Oncology in Southern China, Sun Yat-sen University Cancer Center, Collaborative Innovation Center for Cancer Medicine, Guangzhou 510060, China; 2 Department of Gynecology, Sun Yat-sen University Cancer Center; Collaborative Innovation Center for Cancer Medicine, Guangzhou 510060, China; 3 Department of Chest, Second People's Hospital of Guangdong Province, Guangzhou 510317, China; 4 Department of Pathology, Sun Yat-sen University Cancer Center; Collaborative Innovation Center for Cancer Medicine, Guangzhou 510060, China

**Keywords:** SPAG5, cervical cancer, mTOR

## Abstract

Previously, we found that sperm-associated antigen 5 (SPAG5) was upregulated in pelvic lymph node metastasis–positive cervical cancer. The aim of this study is to examine the role of SPAG5 in the proliferation and tumorigenicity of cervical cancer and its clinical significance in tumor progression. In our study, SPAG5 expression in cervical cancer patients was detected using quantitative real-time polymerase chain reaction, western blotting, and immunohistochemistry; cervical cancer cell function with downregulated SPAG5 *in vitro* was explored using tetrazolium assay, flow cytometry, and colony formation and Transwell assays. SPAG5 was upregulated in tumor tissue compared with paired adjacent noncancerous tissues; SPAG5 upregulation in tumor tissues indicated poor disease-free survival, which was also an independent prognostic indicator for cervical cancer patients. *In vitro* study demonstrated that SPAG5 downregulation inhibited cell proliferation and growth significantly by G2/M arrest and induction of apoptosis, and hindered cell migration and invasion. Under SPAG5 downregulation, the sensitivity of cervical cancer cells differed according to taxol dose, which correlated with mammalian target of rapamycin (mTOR) signaling pathway activity. In general, SPAG5 upregulation relates to poor prognosis in cervical cancer patients, and SPAG5 is a regulator of mTOR activity during taxol treatment in cervical cancer.

Cervical cancer ranks the third frequently diagnosed cancer in women worldwide. Although the morbidity rate has declined slightly over the past decade, cervical cancer remains the second most frequent cause of cancer-related death in women in developing countries.^1^ The causal relationship between human papillomavirus infection and cervical cancer is clear, but the mechanisms of cervical cancer progression are mostly unknown. Pelvic lymph node metastasis (PLNM) is an important prognostic factor for patients with cervical cancer.^2^ Early detection of metastasis at the local or regional lymph nodes is pivotal in planning therapy. Previously, we constructed a 1440 probe-customized oligonucleotide microarray and screened out 32 differentially expressed genes between PLNM-positive and PLNM-negative cervical cancer specimens.^2^ One of these genes, sperm-associated antigen 5 (*SPAG5* or *Astrin*), was upregulated in PLNM-positive specimens compared with PLNM-negative specimens. Considering the vital role of SPAG5 in cell mitosis, we focused on SPAG5 protein in this study to search for its functions in cervical cancer.^3^

Chang *et al.*^4^ first cloned and investigated SPAG5 in 2001 as a mitotic spindle-associated protein. SPAG5 is upregulated in M-phase cells and binds to microtubules as a regulator of the timing of spindle organization and separation of sister chromatids.^3, 5^ Although the specific SPAG5 mechanisms involved in cell cycle regulation are unclear, several cell cycle-related proteins, for example, p29, Aurora-A, and SNM1B, bind to SPAG5 and have a role in DNA damage recovery.^4, 6, 7^ At the same time, the localization of SPAG5 to the centrosome is vital to its function.^8^ It has been demonstrated that SPAG5 can be phosphorylated by glycogen synthase kinase-3*β* and polo-like kinase 1, which are important for SPAG5 activity.^9, 10, 11, 12^ SPAG5 is clearly an important factor in mitosis and cell cycle checkpoint regulation; Thedieck *et al.*^13^ recently reported that SPAG5 downregulated the mammalian target of rapamycin (mTOR) signaling pathway upon oxidative stress and protected cells from apoptosis. This new theory further indicates that SPAG5 is a promoter in tumorigenesis and progression. Nevertheless, only two studies on estrogen receptor-positive breast cancer and non-small-cell lung cancer reported high SPAG5 expression by microarray analysis;^14, 15^ whether SPAG5 expression is related to the etiology and development of cervical cancer remains unclear.

Based on our novel findings and the above mentioned reports, we hypothesized that *SPAG5* is a candidate oncogene in cervical cancer. To gain insight into the clinical roles and biological functions of SPAG5, we investigated its expression in cervical cancer specimens using quantitative real-time polymerase chain reaction (qRT-PCR), western blotting, and immunohistochemistry (IHC), and the effects of SPAG5-targeting small interfering RNA (siRNA) on cervical cancer cell line behaviors by 3-(4,5-dimethylthiazol-2-yl)-2,5-diphenyltetrazolium bromide (MTT) assay, flow cytometry, colony formation assay, and Transwell assay. We also explored the role of SPAG5 in taxol antitumor mechanisms as well as its impact on the mTOR signaling pathway.

## Results

### SPAG5 was upregulated in cervical cancer and contributed to prognosis

**Figure 1:**
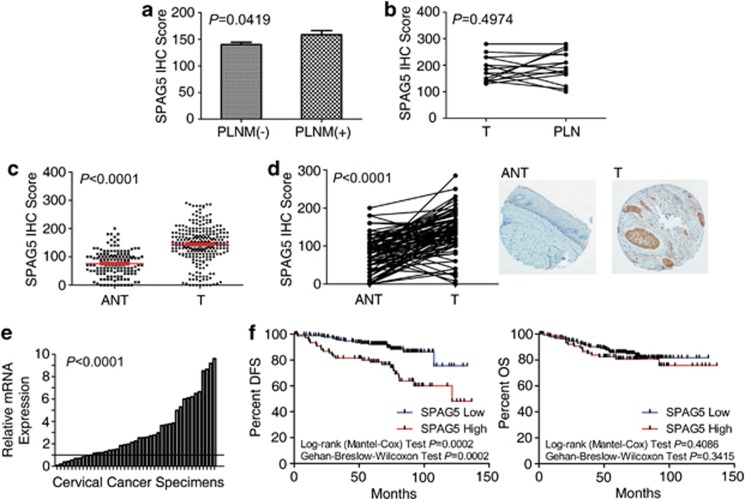
Upregulated SPAG5 in PLNM-positive cervical cancer tissues and cervical cancer tissues indicating poorer prognosis. (**a**) IHC staining indicating higher SPAG5 expression in PLNM-positive specimens. (**b**) No significant differences between the LNM lesion (PLN) and the paired primary tumor (T). (**c**) Protein expression was higher in T tissues compared with ANT in general and in (**d**) paired T tissues (right) with ANT (left; × 40 amplification). (**e**) qRT-PCR confirming *SPAG5* mRNA upregulation in T tissues standardized with paired ANT. (**f**) Disease-free survival (DFS) and overall survival (OS) according to high and low SPAG5 protein expression was compared with Kaplan–Meier plots; DFS was worse with high SPAG5 expression; there was no difference in OS

Via gene profiling using a customized oligonucleotide microarray, we previously reported that SPAG5 was upregulated in PLNM-positive cervical cancer tissues compared with PLNM-negative tissues.^2^ In this study, we investigated 260 cervical cancer tissues and 147 adjacent noncancerous cervical epithelial tissues at protein level with IHC staining. By comparing the IHC staining of 193 PLNM-negative and 64 PLNM-positive tumor tissues, we confirmed that SPAG5 was upregulated in PLNM-positive specimens (*P*=0.0419; Figure 1a). However, there were no significant differences between metastasis and the primary lesion (*n*=15, *P*=0.4974; Figure 1b).

The IHC staining also demonstrated clearly higher SPAG5 protein levels in cervical cancer tissues than in adjacent noncancerous tissues (ANT; *P*<0.0001; Figure 1c). The results from paired tissues also revealed higher SPAG5 protein levels in 78/90 pairs (86.67%) and lower SPAG5 protein levels in 12/90 pairs (13.33% *P*<0.0001; Figure 1d). The *SPAG5* mRNA expression levels in another 40 pairs also demonstrated high *SPAG5* expression in tumors (33/43, 76.73%, paired *t*-test, *P*<0.0001; Figure 1e). Owing to the consistence of SPAG5 mRNA and protein expression, there may be little post-transcriptional regulation of this protein.

To explore the clinical significance of SPAG5 expression in cervical cancer patients, we analyzed the relationship between SPAG5 expression and the patients' clinical data. According to the 170 cutoff score by receiver operator characteristic analysis, which has the best sensitivity and specificity for distinguishing tumors and adjacent noncancerous cervical tissues (*P*=0.006), patients were classified into high and low SPAG5 expression groups with SPAG5 expression in tumor tissues. Based on survival analysis, patients with high SPAG5 expression had poorer disease-free survival (DFS; DFS rate of low expression group: 90.164% high expression group: 68.831% *P*<0.0001), and there were significant differences between the survival curves of these two groups (log-rank test, *P*=0.0002; Gehan–Breslow–Wilcoxon test, *P*=0.0002; Figure 1f, left). The overall survival (OS) rate was slightly higher in the SPAG5 low expression group (low expression group: 86.188% high expression group: 80.519% *P*=0.2497), but there were no significant differences in Kaplan–Meier plots (log-rank test, *P*=0.4086; Gehan–Breslow–Wilcoxon test, *P*=0.3415; Figure 1f, right). Logistic regression analysis indicated SPAG5 expression as one of the independent prognostic factors for DFS (Table 1).

These results indicated that SPAG5 expression in cervical cancer has an important role in both tumorigenesis and tumor progression.

### SPAG5 knockdown impaired cell proliferation and mobility

**Figure 2:**
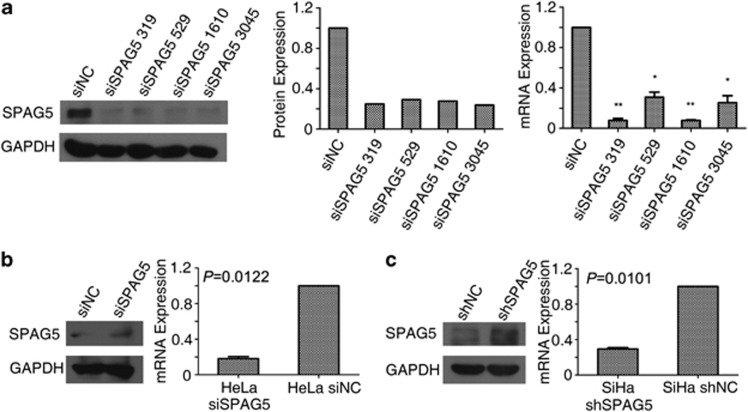
SPAG5 knockdown efficiency in cervical cancer cell lines. (**a**) Western blot of siRNA specific to SPAG5 knockdown efficiency in SiHa cells (left), gradient analysis (middle); mRNA expression (right) was detected by qRT-PCR. (**b**) Transient knockdown reflected in levels of SPAG5 protein (left) and mRNA (right) in HeLa cells. (**c**) SPAG5 expression in shSPAG5 lines detected by western blotting (right) and qRT-PCR (left)

To determine how SPAG5 regulates the biological behavior of cervical cancer cells, we designed four siRNAs targeting *SPAG5* mRNA to eliminate SPAG5 regulation in cervical cancer cells and to study its function. The knockdown efficiency of the siRNAs was detected by qRT-PCR and western blotting (Figure 2a). Owing to its high efficacy, siSPAG5 319 was selected to perform the following experiments, and we will use ‘siSPAG5' directly instead of ‘siSPAG5 319' in the article to make our expression concise. The SiHa shSPAG5 and SiHa shNC cell lines were constructed based on the siSPAG5 319 and negative control (NC) siRNA sequences (Figure 2c).

**Figure 3:**
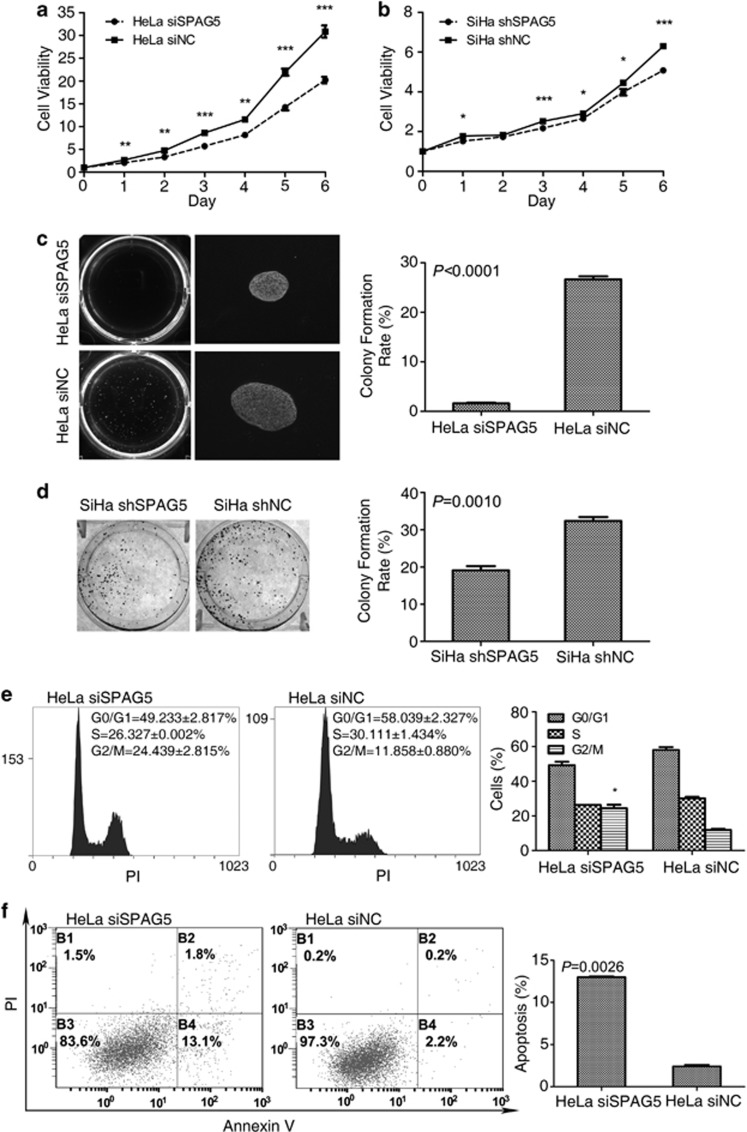
SPAG5 knockdown inhibited cervical cancer cell proliferation. Cell viability analysis of the growth curves of (**a**) transient SPAG5 knockdown HeLa and (**b**) shSPAG5 cells. (**c**) Transient SPAG5 knockdown HeLa soft agar colony showing inhibited colony quantity, size, and formation rates ( × 200). (**d**) SiHa shSPAG5 plate colony formation also showing inhibited colony formation rate. (**e**) G2/M arrest and (**f**) apoptosis induced by SPAG5 downregulation

The growth curves of SPAG5 knockdown cells were determined by MTT assay. The growth of SPAG5 transient knockdown HeLa cells (Figure 2b) and SiHa shSPAG5 cells was significantly inhibited (Figures 3a and b). In the soft agar colony formation assay, the HeLa cell colony formation rate decreased from 26.63 to 1.6% (*P*<0.0001) because of the transient knockdown of SPAG5; additionally, the size of colonies formed by SPAG5 knockdown cells were smaller (Figure 3c). Plate colony formation assay of SiHa shSPAG5 and SiHa shNC cells also demonstrated inhibited colony formation rates. However, because of the poor colony formation ability of SiHa cells, the result was not apparent; with the formation rate decreasing from 32.4 to 19.13% (*P*=0.0010; Figure 3d). To examine the mechanisms involved in inhibition of cell growth and proliferation in SPAG5 knockdown, we analyzed apoptosis and the cell cycle. SPAG5 downregulation induced remarkable G2/M arrest; the proportion of G2/M cells increased from 11.858±0.880% to 24.439±2.815% (*P*=0.0264) without influencing other cell cycle phases (Figure 3e). The rates of apoptosis increased from 2.4±0.283% to 13±0.141% in transient SPAG5 knockdown in HeLa cells (*P*=0.0026; Figure 3f).

**Figure 4:**
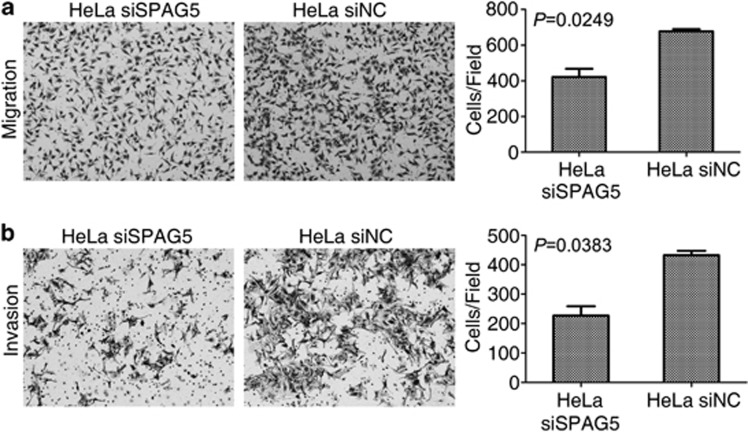
SPAG5 knockdown inhibited cervical cancer cell migration and invasion. (**a**) Transient SPAG5 knockdown inhibited the migration ability of HeLa cells. (**b**) Transwell assay with Matrigel revealing decreased invasion ability following SPAG5 knockdown in HeLa cells

In addition to cell growth and proliferation, cell mobility is also important to tumor severity; therefore, we analyzed the migration and invasion abilities of SPAG5 knockdown HeLa cells. Cells that migrated to the bottom of the Transwell chambers decreased from 667.667±21.079 to 421.333±80.308 per high power field (*P*=0.0249; Figure 4a). The number of cells that invaded through the Matrigel and migrated decreased from 432.333±25.580 to 227.333±54.077 (*P*=0.0383; Figure 4b). We concluded that SPAG5 knockdown suppressed cell mobility.

### SPAG5 expression influenced the antitumor effect of taxol

**Figure 5:**
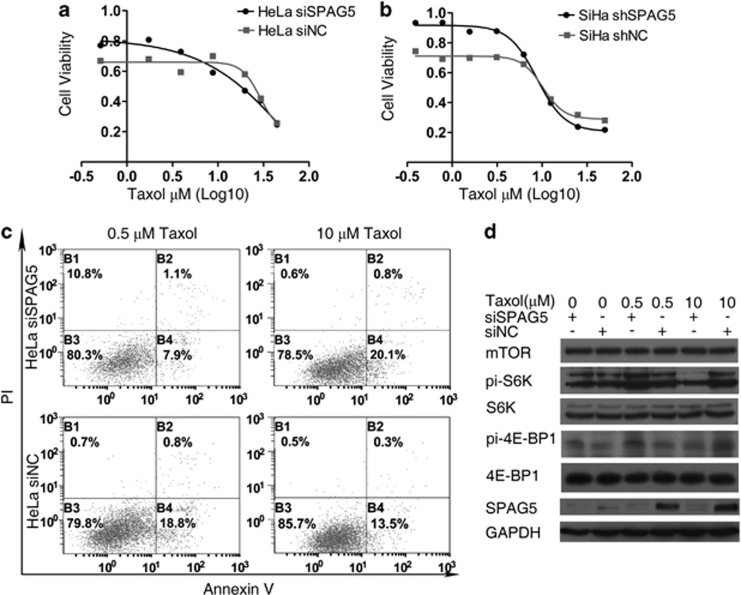
SPAG5 regulation influenced mTOR signaling pathway activation in cervical cancer cell lines under taxol treatment. Taxol treatment after SPAG5 downregulation in (**a**) HeLa and (**b**) SiHa cervical cancer cell lines revealing differing drug sensitivities under different concentrations due to (**c**) apoptosis. (**d**) Western blot analysis of mTOR signaling pathway activation

To test whether SPAG5 downregulation was related to the antitumor effect of taxol, we constructed a taxol concentration gradient and performed the MTT assay to observe the drug sensitivities of SPAG5 knockdown and NC cells (Figures 5a and b). At 0.5 *μ*M (low dose), SPAG5 transient knockdown HeLa were cells tolerant of taxol. However, at 10 *μ*M (high dose), SPAG5 knockdown sensitized cells to taxol. The same observation was made in SiHa shSPAG5 cells. Furthermore, we examined taxol-induced apoptosis under SPAG5 regulation; the results were in accord with that of the taxol inhibition analysis. The proportion of apoptotic cells decreased from 18.8 to 7.9% when treated with 0.5 *μ*M taxol under SPAG5 knockdown compared with the NC. Conversely, in SPAG5-downregulated cells treated with 10 *μ*M taxol, the rate of apoptosis increased from 13.5 to 20.1% (Figure 5c).

The mTOR signaling pathway regulates cell growth and apoptosis and is associated with SPAG5 and taxol treatment.^13, 16^ MTOR signaling pathway activity in HeLa cells was examined by western blotting (Figure 5d). SPAG5 protein levels were elevated by taxol treatment and were concentration dependent, and were all downregulated significantly by SPAG5-targeting siRNA. S6K and 4E-BP1 phosphorylation were increased following SPAG5 knockdown. Neither SPAG5 downregulation nor taxol treatment altered mTOR, S6K, or 4E-BP1 expression. Taxol activated the mTOR signaling pathway; the levels of activation were inversely correlated with apoptosis, but SPAG5 downregulation altered activation. When SPAG5 was downregulated, 0.5 *μ*M taxol could activate the mTOR pathway compared with the NC; in contrast, 10 *μ*M taxol inhibited mTOR pathway activation. MTOR pathway activation induced apoptosis in HeLa cells following SPAG5 downregulation. However, the mTOR signaling pathway protected the HeLa cells from apoptosis under taxol treatment, and SPAG5 regulated the activity of mTOR signaling, which influenced apoptosis.

In general, the mTOR signaling pathway under taxol treatment has an opposite role in apoptosis compared with the absence of taxol. In the meantime, SPAG5 downregulation can alter mTOR pathway activity.

## Discussion

Much effort has been expended on the primary, secondary, and tertiary prevention of cervical cancer over the decades, but more work is still required to develop therapeutic strategies against cervical cancer. Previously, we found that SPAG5 was upregulated in PLNM-positive tumor tissues: 15/23 PLNM-positive patients had high SPAG5 expression (65.217%) compared with 4/64 PLNM-negative patients (6.154%).^2^ Two other microarray profiling studies mentioned the SPAG5 upregulation in estrogen receptor-positive breast cancer and non-small-cell lung cancer,^14, 15^ but there have been no specific studies. In our recent study, we confirmed that SPAG5 protein was elevated in PLNM-positive patients and clearly upregulated in tumor tissues compared with paired ANT. Moreover, high SPAG5 expression was associated with lower DFS rate and shorter DFS period. High SPAG5 expression was an independent prognostic factor for cervical cancer patients. Combining the function of SPAG5 in cell spindle construction and mitosis with our findings, we believe that SPAG5 contributes to cervical cancer progression.

Cell biological behaviors under SPAG5 downregulation were tested by RNA interference. SPAG5 downregulation in cervical cancer cell lines inhibited cell viability and growth. Analysis of the cell cycle and apoptosis primarily clarified the reasons for inhibition of cell growth and proliferation by SPAG5 downregulation. In accord with the ability of SPAG5 to maintain and promote mitosis, its downregulation induced significant G2/M arrest. Apoptosis was also increased that might be the consequence of cell cycle deregulation and mitotic catastrophe.^3, 5^ Cell migration and invasion were also inhibited in SPAG5-downregulated cells, which may be attributed to the microtubule-binding ability of SPAG5.^17, 18^ These results demonstrated that SPAG5 has a significant role in both tumorigenesis and progression.

In view of the above results, we assumed that SPAG5 downregulation exerted an antitumor effect. To understand how this was accomplished, we explored the role of SPAG5 in taxol treatment. Taxol is one of the most important first-line treatments in cervical cancer chemotherapy, inhibiting the disassembly of microtubule polymers.^19, 20^ Taxol inhibits cell spindle assembly, mitosis, and eventually induces apoptosis, which is more likely the consequence of SPAG5 downregulation. Under taxol treatment, SPAG5 expression was elevated because of the G2/M arrest. Previous study found that SPAG5 was a protective agent in cells under oxidative stress via the recruitment of raptor to stress granules and downregulation of mTOR complex 1 (mTORC1) activity.^13^ When cells are stressed, mTOR overactivation induces apoptosis. Considering taxol and mTOR signaling together, taxol activated phosphoinositide-3-kinase (PI3K)/mTOR signaling.^21^ Moreover, several studies have reported synergy between mTOR inhibitors and taxol.^16, 22, 23, 24, 25^ The anti-human epidermal growth factor receptor type 2 (HER2) antibody trastuzumab downregulates Akt/PI3K activity and suppresses SPAG5 expression when combined with taxol.^26^ Considering the present findings and the above mentioned reports, we believe that SPAG5 influences the taxol therapeutic effect by modifying mTOR activity.

Treatment using different concentrations of taxol may generate different cell biological behaviors. We treated SPAG5 knockdown and NC cervical cancer cells with different doses of taxol and identified the different roles of SPAG5 under these doses. Analysis of apoptosis confirmed that SPAG5 promoted apoptosis under low-dose taxol but protected cells from apoptosis under high-dose taxol. Searching further for the related mechanisms, we found that mTOR signaling pathway activity has an important role in this process. Where mTOR activation promoted apoptosis, SPAG5 downregulation elevated mTOR activity and induced apoptosis. In contrast, mTOR activation protected cells from apoptosis under taxol treatment. Under these circumstances, the effects of taxol may rely on autophagy rather than apoptosis.^27^ SPAG5 downregulation altered mTOR activity and influenced the eventual apoptosis. We concluded that SPAG5 exerts a vital moderating effect on taxol treatment.

SPAG5 was not only an independent prognostic factor for cervical cancer patients, but also had an important role in the effects of chemotherapy. mTOR signaling pathway inhibitors might be a new therapeutic treatment in cervical cancer,^28^ and SPAG5 expression in cervical cancer may be a potential indicator for mTOR inhibitor treatment.

## Materials and Methods

### Human samples

Human samples comprising cervical cancer tissues and ANT were obtained from surgery patients of initial treatment at Sun Yat-Sen University Cancer Center (SYSUCC), Guangzhou, China, between 2001 and 2009. Paraffin-embedded samples from 297 specimens and 43 paired specimens (tumor tissues and ANT) treated with RNAlater (Life Technologies, Carlsbad, CA, USA) followed by liquid nitrogen refrigeration were collected. Written informed consent was obtained before the investigation. Staging was according to the International Federation of Gynecology and Obstetrics (FIGO 1994) classification guidelines; an experienced pathologist graded and subtyped specimens histopathologically based on World Health Organization criteria. Patients attended follow-up visits regularly. The SYSUCC Medical Ethics Committee approved the study protocol.

### Immunohistochemistry

We examined 260 cervical cancer and 147 adjacent noncancerous paraffin-embedded tissues, which included 90 paired specimens, and constructed tissue microarrays. We selected 14 more paired lymph node tissues with metastasis from the 297 specimens. IHC staining of the 260 cervical cancer tissues was analyzed with their clinical data. Among the 260 cervical cancer specimens, 200 were >35 years old and 60 were ≤35 years old; 56 were FIGO Stage IIA/IIB and 204 were in FIGO Stage IB1/IB2. IHC staining was performed after dewaxing, 10-min incubation with 0.3% H_2_O_2_ to block endogenous peroxidase, and 25-min microwave antigen retrieval with SPAG5 antibody (1 : 400; Sigma-Aldrich, St. Louis, MO, USA) at 4 °C overnight. This was followed by horseradish peroxidase (HRP)-labeled secondary antibody incubation and diaminobenzidine chromogenic reaction in a GT Vision III Detection System/Mo Rb (GeneTech, Shanghai, China). Cytoplasmic SPAG5 staining was scored by staining intensity (0, negative; 1, weakly positive; 2, moderate; 3, strongly positive) multiplied by the percentage of corresponding staining area.

### Quantitative real-time PCR

The 43 paired specimens of cervical cancer were treated with RNAlater (Life Technologies) overnight at 4 °C and transferred to a nitrogen storage refrigerator. Cultured cells were digested by 0.25% trypsin–ethylenediaminetetraacetic acid (Life Technologies) and washed with phosphate-buffered saline (PBS) twice before homogenizing. Total RNA was extracted using TRIzol (Life Technologies) according to the manufacturer's instructions. Relative SPAG5 mRNA expression levels were analyzed by qRT-PCR with double-stranded DNA-binding dyes as reporters. GoTaq qPCR Master Mix (Promega, Madison, WI, USA) was used and glyceraldehyde-3-phosphate dehydrogenase (GAPDH) mRNA expression was used as the internal reference. qRT-PCR primers were SPAG5 forward: 5′-CATCTCACAGTGGGATAACTAATAAAC-3′, SPAG5 reverse: 5′-CAGGGATAGGTGAAGCAAGGATA-3′ GAPDH forward: 5′-CTCCTCCTGTTCGACAGTCAGC-3′, GAPDH reverse: 5′-CCCAATACGACCAAATCCGTT-3′.

### Cell lines

The human cervical cancer cell lines HeLa and SiHa and the human kidney cell line HEK-293T were cultured in Dulbecco's modified Eagle's medium (DMEM; Life Technologies) supplemented with 10% fetal bovine serum (FBS; Life Technologies). Cell lines were incubated at 37 °C with 5% CO_2_ and 95% humidity. All cells were obtained from Cell Bank, Shanghai Institutes for Biological Sciences (Shanghai, China).

SiHa with SPAG5 stable knockdown (SiHa shSPAG5) cell line and NC cell line (SiHa shNC) were constructed by infection with retrovirus carrying short hairpin RNA (shRNA) sequence specifically targeting SPAG5, and scramble sequence as the NC respectively. At 48 h after infection, 0.3 *μ*g/ml puromycin (EMD Millipore, Billerica, MA, USA) was added to the culture medium to select for positive clones. The virus packing and infection procedure, which used a pSUPER RNAi System (OligoEngine, Seattle, WA, USA), was performed according to the manufacturer's directions. SiHa shSPAG5 and SiHa shNC cell lines were constructed after 3 weeks of screening and confirmed based on SPAG5 mRNA and protein expression levels by qRT-PCR and western blotting, respectively.

### Western blotting

Protein was extracted with ProteoJET Mammalian Cell Lysis Reagent (Thermo Fisher Scientific, Waltham, MA, USA) supplemented with protease inhibitor. Protein samples were treated with Dual Color Protein Loading Buffer (Thermo Fisher Scientific) containing reducing agent at 100 °C for 5 min, resolved on 10% Tris-HCl polyacrylamide gels, and transferred to a polyvinylidene fluoride membrane. Overnight incubation (4 °C) of the primary antibody was followed by HRP-conjugated anti-rabbit (1 : 1000; Novus Biologicals, Littleton, CO, USA) or anti-mouse antibody (1 : 1000; Novus Biologicals) and Pierce ECL Western Blotting Substrate (Thermo Fisher Scientific). Antibody dilution was as follows: SPAG5, 1 : 300 (Sigma-Aldrich); mTOR, 1 : 1000 (Cell Signaling, Danvers, MA, USA); S6K, 1 : 1000 (Cell Signaling); phosphorylated (pi)-S6K (Thr 389), 1 : 1000 (Cell Signaling); 4E-BP1, 1 : 500 (Cell Signaling); pi-4E-BP1 (Thr 37/46), 1 : 1000 (Cell Signaling); and GAPDH, 1 : 1000 (Abgent, San Diego, CA, USA).

### MTT assay

MTT (MP Biomedicals, Shanghai, China) powder was dissolved to 5 mg/ml in double-distilled water and filtered with a 0.22-*μ*m polysulfone membrane filter. The final MTT concentration was 0.5 mg/ml when performing the assay. After 4-h incubation, the medium was discarded and 150 *μ*l dimethyl sulfoxide (MP Biomedicals) per well of a 96-well plate was added, followed by detection of optical density at 490 nm.

### Cell cycle analysis

Before the assay, cells were synchronized by 24-h culture without FBS, and then cultured in complete medium for 20 h. Cultured cells were digested with 0.25% trypsin, washed twice with PBS, and fixed with 75% ethanol that had been precooled at −20 °C for 1 h. Samples were centrifuged to discard the ethanol and suspend with the PBS before RNase A (0.2 mg/ml) treatment at 37 °C for 30 min. DNA was stained with propidium iodide (PI; 50 *μ*g/ml) for 30 min on ice away from light, and the cell cycle was analyzed by flow cytometry.

### Detection of apoptosis

Apoptosis was detected by annexin V–fluorescein isothiocyanate (FITC) and PI double staining with an Annexin V-FITC Apoptosis Detection Kit (KeyGen, Nanjing, China). Cells were digested using 0.25% trypsin (Life Technologies) and washed twice using PBS. Binding buffer was added to the resuspended cells, which were then stained with annexin V-FITC/PI at room temperature for 15 min away from light. Apoptosis was analyzed by flow cytometry.

### Taxol treatment

SPAG5 was transiently knocked down in HeLa cells 24 h before taxol (Sigma-Aldrich) treatment. HeLa SPAG5 transient knockdown and SiHa shSPAG5 cells were treated with 0, 0.5, or 10 *μ*M taxol for 24 h; cell viability was analyzed using the MTT assay.

### Colony formation assay

Plate colony formation assay was performed by seeding 1000 cells/well into six-well plates in triplicate. Fresh complete medium was changed every 5 days. Ten days after seeding, the medium was discarded and cells were dyed using 0.5% crystal violet in methanol (Sigma-Aldrich).

Soft agar colony formation was performed using low-melting point agarose (Sigma-Aldrich). A base layer of 2 ml 10% FBS/DMEM containing 0.7% agarose was spread in a six-well plate; 1 × 10^3^ cells were resuspended in 1 ml 10% FBS/DMEM containing 0.35% agarose as the upper layer. Plates were incubated at 37 °C with 5% CO_2_ for 20 days.

### Transwell assay

The Transwell assay was performed to analyze the metastasis ability of the cells. BD Falcon Cell Culture Inserts with 8.0-*μ*m pores (BD Biosciences, Franklin Lakes, NJ, USA) were placed in a 24-well plate with 600 *μ*l 10% FBS/DMEM in the lower chamber. To test migration ability, 1 × 10^5^ cells suspended in 100 *μ*l FBS-free DMEM were seeded directly into the upper chamber. After 15-h culture, cells that had migrated to the bottom of the chamber membrane were dyed with 0.5% crystal violet (Sigma-Aldrich) dissolved in methanol. To test invasion ability, 5 × 10^5^ cells suspended in 100 *μ*l FBS-free DMEM were seeded into the upper chamber pre-coated with 50 *μ*l BD Matrigel Basement Membrane Matrix (BD Biosciences) diluted 1 : 2 with FBS-free DMEM. After 20-h incubation, cells were dyed as mentioned above.

### Transient transfection

Four siRNAs targeting different *SPAG5* mRNA sites were designed: siSPAG5 319 sense: 5′-CAGACUUAUCUUCAGAACATT-3′, siSPAG5 319 antisense: 5′-UGUUCUGAAGAUAAGUCUGTT-3′ siSPAG5 529 sense: 5′-AGGCCCGUUUAGAUACCAUTT-3′, siSPAG5 529 antisense: 5′-AUGGUAAUCUAAACGGGCCUTT-3′ siSPAG5 1610 sense: 5′-GGGUGCUUAUCUCUAAAGATT-3′, siSPAG5 1610 antisense: 5′-UCUUUAGAGAUAAGCACCCTT-3′, siSPAG5 3045 sense: 5′-CCUGCUACAAGAGUCUAAATT-3′, siSPAG5 3045 antisense: 5′-UUUAGACUCUUGUAGCAGGTT-3′. Scramble sequence was used as the NC: siNC sense: 5′-UCCUCCGAACGUGUCACGUTT-3′ and siNC antisense: 5′-ACGUGACACGUUCGGAGAATT-3′. SiRNA mimics were transfected to target cells with Lipofectamine RNAiMAX (Life Technologies) according to the manufacturer's directions. The small interference sequence was constructed as shRNA and inserted into pSUPER.retro.puro. Plasmids facilitating virus packaging were cotransfected to HEK-293T with Lipofectamine 2000 (Life Technologies) according to the manufacturer's direction.

### Statistical analysis

Data reported by bar or column are depicted as mean±S.E.; all experiments were performed in triplicate. Statistical analysis was performed using PASW Statistics 18 (IBM, Armonk, NY, USA); differences were assessed with an unpaired or paired two-tailed *t*-test, log-rank (Mantel–Cox) test, and the Gehan–Breslow–Wilcoxon test for Kaplan–Meier plots, or by the *χ*^2^-test. *P*-values are depicted directly in graphs or considered as follows: *P<0.05 (significant), **P<0.01 (very significant), and ***P<0.001 (highly significant).

## Figures and Tables

**Table 1:** Logistic regression analysis of factors associated with disease-free survival

**Clinical variable**	**Subset**	**HR (95% CI)**	***P-*****value**
*Univariate analysis,* n=*260*			
Age, years	>35 *versus* ≤35	0.681 (0.352–1.316)	0.2526
FIGO stage	IIA/IIB *versus* IB1/IB2	1.375 (0.691–2.737)	0.3643
Grade of differentiation	1, 2, 3	1.901 (1.042–3.471)	0.0363^a^
Greatest tumor dimension, cm	>4 *versus* ≤4	0.922 (0.421–2.020)	0.8388
Lymphovascular space invasion	Yes or no	1.714 (0.605–4.859)	0.3107
Depth of cervical invasion	≥66% *versus* <66%	1.892 (0.944–3.793)	0.0722
Uterine corpus invasion	Yes or no	2.218 (1.086–4.530)	0.0288^a^
Pelvic lymph node metastasis	Yes or no	2.236 (1.207–4.141)	0.0105^a^
SPAG5 expression	High *versus* low	3.030 (1.638–5.605)	0.0004^a^

*Multivariate analysis,* n=*260*			
SPAG5 expression	High *versus* low	3.589 (1.796–7.173)	0.0003^a^
Grade of differentiation	1, 2, 3	1.614 (0.832–3.132)	0.1568
Uterine corpus invasion	Yes or no	2.029 (0.965–4.269)	0.0622
Pelvic lymph node metastasis	Yes or no	2.623 (1.345–5.115)	0.0047^a^

Abbreviations: CI, confidence interval; HR, hazard ratio

a *P*-value with significance (*P*<0.05)

## Abbreviations

ANT: adjacent noncancerous tissues
DFS: disease-free survival
DMEM: Dulbecco's modified Eagle's medium
FIGO: International Federation of Gynecology and Obstetrics
GAPDH: glyceraldehyde-3-phosphate dehydrogenase
HRP: horseradish peroxidase
IHC: Immunohistochemistry
MTT: 3-(4,5-Dimethylthiazol-2-yl)-2,5-diphenyltetrazolium bromide
OS: overall survival
mTOR: mammalian target of rapamycin
PBS: phosphate-buffered saline
PI: propidium iodide
PI3K: phosphoinositide-3-kinase
PLNM: Pelvic lymph node metastasis
qRT-PCR: quantitative real-time polymerase chain reaction
siRNA: small interfering RNA
SPAG5: sperm-associated antigen 5
SYSUCC: Sun Yat-Sen University Cancer Center
